# Lessons learned implementing an innovative extension for community healthcare outcomes (ECHO) program

**DOI:** 10.3389/frhs.2025.1682447

**Published:** 2026-01-12

**Authors:** Sally Kraft, Megan Colgan, Heather Carlos, Seddon Savage

**Affiliations:** 1 Department of Population Health, Dartmouth Health, Lebanon, NH, United States; 2 The Dartmouth Institute for Health Policy and Clinical Practice, Geisel School of Medicine at Dartmouth, Hanover, NH, United States; 3 Geisel School of Medicine at Dartmouth, Hanover, NH, United States; 4 Dartmouth Cancer Center, Lebanon, NH, United States

**Keywords:** community education, ECHO, implementation frameworks, innovation, population health

## Abstract

As the United States faces mounting challenges to improving health outcomes, new strategies are needed to address root drivers of health and engage community partners to change the community conditions that impact health and health disparities. Project ECHO (Extension for Community Healthcare Outcomes) is a telementoring model developed in 2003 at the University of New Mexico to disseminate knowledge, share evidence-based care practices, and create communities of learning. The ECHO model has been shown to improve clinical outcomes by training primary care care clinicians to provde care often delegated to specialists. This paper describes modifications to ECHO programming to improve population health through engagement of diverse, community audiences in order to impact non-clinical contributors to health. During these community-facing ECHO courses, participants learn from short didactic sessions, share best practices through case-based presentations, and increase connections between sectors of the community and the health system. Implementation of this novel ECHO program is described using the RE-AIM and CFIR frameworks. Adapting the ECHO model to support collaborative learning to impact upstream drivers of health may be an important innovation for improving population health.

## 1 Introduction

Over the past decade, the health and wellbeing of the United States population has declined. Despite spending more on health care than any other country, US life expectancy and measures of morbidity demonstrate a growing US health disadvantage compared to other countries (1, 2). Furthermore, US health outcomes vary widely across geographies and between populations based on race, ethnicity and socioeconomic status (3, 4). These patterns of declining US health status have prevailed for decades but were highlighted by the COVID-19 pandemic (3, 5–7).

To improve population health, efforts have to include a focus on the “upstream” drivers of health (8–12). Social determinants of health—the conditions in which people are born, live, work and age and people's access to power, money and resources (13)—are increasingly recognized as important drivers of health and health systems are increasingly incentivized to screen patients for social risks (14). Insight into the etiologies of community conditions include discussions related to the political and structural determinants of health (15). Health systems cannot solve the complex web of inter-related causes of poor health but they must be part of the solution.

Health systems need to collaborate with and activate communities, building community capacity to change the socio-economic-political drivers of health and the forces that create health and health disparities (16–18). New models of learning need to be developed. Academic health systems, responsible for training the health care workforce of the futures, can adapt successful clinical education and training models to build community partnerships for collaborative learning and action.

The Extension for Community Healthcare Outcomes (ECHO) is a telementoring education model to disseminate clinical knowledge and increase community physician's capacity for treatment of complex disease. Developed at the University of Mexico, ECHO programs bring specialized knowledge to primary care physicians, increasing workforce capacity in rural and underserved areas (19). ECHO programs have been replicated across the globe, proving to be a scalable model to address a wide range of health topics.

Dartmouth Health (DH) launched its ECHO program hub in 2019 and quickly broadened its ECHO portfolio from traditional clinically oriented ECHO courses to the development of ECHO courses designed to engage the broader community in addressing the complex, upstream forces that impact population health and health equity. DH ECHO community-focused courses are co-created with non-clinical experts from the community, incorporating a wide range of knowledge, skills and expertise to educate about diverse issues impacting health, such as state policy, social drivers of health (food, transportation, housing, social isolation, family supports), and public health threats such as vaccine hesitancy and climate change.

In this paper we apply implementation and dissemination frameworks to describe how we adapted traditional clinical ECHO programming to engage the broad community in education about social and political drivers of health. As health systems expand efforts to improve health outcomes traditional education models may be modified to educate and engage communities, supporting partnerships to address the upstream conditions that impact health.

## 2 Context

Dartmouth Health (DH) is the only academic health system in New Hampshire (NH). The system includes the academic medical center, six community or critical-access hospitals located in NH and Vermont (VT), several large, multi-disciplinary ambulatory clinics, a specialized hospital for mental health and addiction therapies, and a visiting nurse and hospice service. DH is the largest provider of health care services in NH and second largest in VT. DH is closely affiliated with the Geisel School of Medicine at Dartmouth which has a long history of purposefully engaging medical students with rural community organizations to improve health.

DH Department of Population Health was organized in 2017 with the goal of improving health and health equity through the intentional alignment of health care delivery transformation and investments in community health. DH Population Health frames its scope of work using the salient stream metaphor: work to improve care delivery in clinics and hospitals (downstream), connecting patients with community resources (midstream), and improving community conditions such as increasing food supports, transportation solutions, affordable housing stock, successful employment, and policies that support healthy lives (upstream) (9).

DH Population Health performs community health needs assessments which provide in-depth insights into barriers to optimal health for the whole population where hospitals are located, assessing needs beyond the patient population receiving care at our facilities (20). Intentional work to reach underserved and historically marginalized populations during assessments provides important information about health disparities in the communities served by each hospital.

In 2019, members of the DH Population Health team attended training at the University of New Mexico ECHO Institute, returning to NH to establish the DH ECHO Hub. The ECHO Hub refers to the infrastructure (people, processes, technology) that produces DH ECHO courses. Each course consists of a set number of sessions (usually 6–10), delivered with regular frequency over a defined period of time and offered without charge. Courses are routinely evaluated with pre- and post-course surveys that evaluate participants reported change in confidence with respect to specific learning objectives, changes in their sense of professional isolation, and asks participants about any anticipated practice changes they will adopt as a result of ECHO participation. A 3-months post-course survey inquiries about actual practice change as a result of ECHO participation. Course topics are identified by a gap in health care service performance, faculty or community members requesting the opportunity to produce an ECHO course, or a community health need.

The DH ECHO Hub team includes physicians with clinical, education, and public health expertise, a nurse, and project managers, who are all embedded in the day-to-day work of Population Health and are committed to addressing the complex interplay between clinical and non-medical drivers of health. The ECHO program infrastructure is supported through a combination of committed dollars from the health system and variable funds from numerous grants and contracts.

Initial programming in 2019 replicated the “traditional” ECHO model with DH specialists sharing knowledge and best practices with primary care teams. With the onset of the pandemic in 2020, the DH ECHO program rapidly pivoted to provide courses designed for clinicians and non-clinical community leaders in order to meet urgent needs associated with the pandemic. Clinical COVID-19 courses were initially produced to support clinical care delivery in primary care and obstetrics. In response to the need to disseminate COVID-19 information to non-clinical audiences, ECHO courses were produced for community health workers to support their work assisting clients with social care needs, and for business owners and employers to provide up-to-date information on practices to keep employees and customers safe and businesses open. These early COVID-19 relevant community-focused ECHOs set the stage for future community-focused ECHOs addressing social and policy drivers of health.

## 3 Defining the community-focused ECHO

The DH ECHO Hub defines community-focused ECHO courses as courses primarily focused on non-clinical topics, including those socio-economic-behavioral and political drivers of population health. Community-focused courses are produced with the broad goal of improving population health and decreasing health disparities.

Unlike traditional clinical ECHOs designed and delivered for clinical audiences, the curriculum for community-facing courses are largely developed by non-clinical subject matter experts from the community and target audiences are largely made up of non-clinicians who are in a position to influence health and social drivers of health in the broader community. Professional titles or job roles do not necessarily distinguish these experts; instead, they are often subject champions, thought-leaders, persons with lived experience, and others who have influence–formally or informally—in the community. At least one Dartmouth faculty member with relevant expertise participates in curriculum development and during course sessions to assure alignment with medical and scientific principles. Additional adaptations of community focused ECHOs include broadening of the definition of “case” from clinical case presentations, to include a variety of other devices to stimulate discussion and the focus on topics that drive health at the community level rather than topics concentrated on clinical care of patients.

Despite program adaptations from traditional ECHOs, the community-focused ECHOs developed at DH share the four key principles of the ECHO model: (1) use technology to leverage scarce resources; (2) share practices to reduce disparities; (3) use case-based learning; and (4) monitor program outcomes (21, 22). Regardless of adaptations implemented in DH community-focused ECHOs, adherence to these principles reinforces core tenets of the original ECHO model.

Since 2019, the number of community-focused ECHOs produced each year has increased, totaling 36 by July 2025, which represent 50% of the total 70 courses produced. The types of courses and participant numbers are displayed in Table 1. Multiple ECHO courses may address a common general topic, e.g., youth mental health, but the courses usually address different aspects of the topic and have different curricula and/or target audiences. Even courses that are repeated with the same title, e.g., the Political Drivers of Health, are continuously modified in response to changes in the environment (current year's bills and State legislature issues in the case of the Political Drivers of Health) and in response to prior course evaluations. Registration is completed online using the same technology platform for all ECHO courses, clinically- and community-focused.

**Table 1:** Community-focused ECHO courses produced from August 2019 to June 2025.

Category	Intended Audience	Courses	Registrants
Youth Mental Health	Community/Schools	13	1,620
COVID-19	Employers/CHWs	6	567
Substance Use Disorder	Employers	4	714
Political Drivers of Health	Community	4	893
Vaccine Hesitancy	Community	3	341
Social Drivers of Health	Librarians	2	193
Community Engagement	Researchers	1	98
Hoarding	Community	1	137
Rural health equity	Community	1	191
Cancer Survivorship	Physical Trainers	1	63
	Total	36	4,817

## 4 Implementation frameworks and community-focused ECHOs

The DH ECHO Hub did not proactively utilize formal implementation science to transition traditional clinical ECHO programs to non-clinical, community-focused programs, however implementation frameworks provide useful structures to describe our course adaptations and inform others interested in replicating these programs.

There is a large literature evaluating the implementation of traditional ECHO and ECHO-like models and these implementation principles are resonant with the evolution of our community-facing programs (21–31). Both the RE-AIM and CFIR frameworks have been used to evaluate traditional ECHO models of education (22, 30) and align well with our experience designing and implementing community-focused ECHOs.

A high-level process map describes the production of a DH ECHO community-facing course from idea generation through course evaluation and maps domains from the CFIR and RE-AIM model to these process steps (Table 2). The RE-AIM framework can be useful for ECHO program planning but it has been noted to have less utility describing the conditions that may impact successful implementation (26). The CFIR framework delineates determinants that impact implementation efforts (32). Combining both frameworks may provide more robust guidance to implementing sustainable programs (26). How these frameworks apply to planning and production of our community-focused ECHOs is described below.

**Table 2:** A high-level process model describing the production of a DH ECHO community-facing course from idea generation through course evaluation mapped to CFIR and RE-AIM domains.

Process Model—high level steps for a proposed ECHO topic					
Processes	Course selection, assess fit with ECHO model	→ Prioritization	→ Planning	Course → Production	Evaluation, → reflection
**Implementation Model**					
**RE AIM**		**Reach**—identify key audience, number of potential participants **Maintenance**— assess degree of alignment with DH Pop Health mission and goals to nurture community-health system relationships	**Adoption**—planning team comprises diverse roles including persons with lived experience **Reach**—planning team members market through their existing networks and DH ECHO team markets to prior ECHO participants	**Implementation**–standard DH ECHO Hub processes including posting course materials on the DH ECHO website at the conclusion of the course	**Effectiveness**—course surveys assess impact on individual participants as measured by self-report. DH ECHO team reviews registration numbers, self-identified participant roles, and attendance at sessions
**CFIR**	**Innovation domain—**proposed community-facing course adapts traditional, clinically-oriented ECHO model to meet goals of improving population health focusing on drivers of health beyond health care services. DH ECHO team evaluates fit with ECHO model	**Inner setting domain**—utilizes DH Pop Health infrastructure and engaged leaders **Individual domain-** available, committed DH ECHO operations team **Outer setting domain** –course will address a current gap in knowledge or need in community	**Implementation domain**—conduct planning team meetings, co-create learning objectives, co-design course sessions **Individuals**—planning team composition includes diverse roles, lived experience, and DH ECHO team staff who facilitate course development	**Implementation domain** –utilize established DH ECHO Hub implementation processes including clear onboarding and technology support for participants, skilled facilitation, case-based learning to apply knowledge, and collaboratively identify best practices	**Implementation domain**—planning team and DH ECHO team meet after course completion to reflect on the course and review surveys and participant input. Lessons learned are incorporated into future course planning and production

Implementation domains are indicated in bold font.

### 4.1 Selection of course topics and fit with ECHO model

DH community-focused ECHO courses are initiated by two basic routes: (1) the DH ECHO team may identify a community-based need and recruit content experts to work with us to develop a proposal; or (2) an individual or group of content experts can propose a course. Community health needs assessments are important sources of information for the ECHO team and DH Population Health colleagues share their deep knowledge of health challenges experienced by patient populations and the broader community with the DH ECHO team, surfacing important topics for potential courses. An online form, prominently posted at our Project ECHO website invites community members, faculty members, or other to propose an ECHO course and provide information on the proposed topic, describe learning objectives, and target audience. Ideas for courses are always discussed with the person proposing the course to further understand the need and the intent of the course.

All proposed courses are evaluated to determine if they can be delivered with fidelity to the evidence-based ECHO model, adhering to the four principles described above. Frequently the decision to use an ECHO format centers on the ability to use interactive, case-based learning and the feasibility of finding case presentations, or proxy activities, to stimulate interactive discussion. Additionally, fit with the ECHO model is determined by the volume of didactic material in a session; ECHO programming minimizes the didactic portion of the session and leverages case-based learning and discussion amongst participants to create a shared learning experience. If the DH ECHO team does not feel the ECHO format is the best learning modality, alternative suggestions are suggested, e.g., webinar or podcast.

### 4.2 Prioritization

Community-focused ECHO proposals are reviewed by the DH ECHO operational team and scored using a prioritization formula based on a number of variables, including: alignment with our health system's annual community needs assessment and improvement plan; resonance with long-term priorities of DH Population Health and our health system; documentation of knowledge gaps; potential population impact; timeliness; availability of requisite expertise; and others (Table 3). This provides an important methodical assessment given constrained resources. The final decision to produce a course may be influenced by a variety of other factors but the prioritization criteria helps align course offerings to meet health system and community needs.

**Table 3:** Scorecard for prioritizing ECHO proposals.

Proposed Course Topic:	
Course Variables	Rating 2-High, 1-Medium, 0-Low, n/a-not applicable
Availability of ECHO resources	
ECHO Coordinator available	
Facilitator available	
Timeframe doable for planning	
DH or external Assets to support the ECHO	
Committed Course or Co-Director for program from D-H or Dartmouth College	
*Physician Director available*	
Coordinator from requesting party	
Accessibility of expert speakers	
*Accessibility of expert panelists*	
Potential funding	
Potential partners	
*Contextual issues related to topic*	
Demonstrated community/population need	
Current gap in knowledge/practice	
Number of potential participants	
Timeliness/urgency	
*Resonance with DH ECHO program goals*	
Improve rural health	
Advance interprofessional/interdisciplinary/team-based care	
ECHO model to spread knowledge	
*Resonance with Population Health goals*	
Advance Health Equity	
Work collaboratively with communities to improve the conditions that impact health	
Partner across DH for a fair and just healthcare system	
TOTAL	

DH ECHO prioritization criteria (Table 3) align with a number of implementation framework constructs. Identification of key audience and potential size of that audience are described as reach in the RE-AIM framework (26). Alignment of proposed courses with the long-term DH Population Health goal to create health system-community partnerships is consistent with the intent to maintain improvements as described by the maintenance element in the RE AIM model.

Prioritization criteria also include elements of CFIR framework domains: the inner setting (DH Population Health department infrastructure); individual domains (existing DH ECHO Hub team and supporting DH staff); and outer setting (community health needs assessments providing information on needs in the community). Particularly important is the assessment of how well a proposed course would meet a community health need by providing education related to a high-need topic.

### 4.3 Planning

Once a community-focused course is approved for production, design and production activities are co-created with community input. Course directors, who are responsible for course development, may be community members or DH faculty or staff; often there are course co-directors with one of each. A planning team is brought together whose members may include professional and/or non-professional persons and every ECHO course strives to include persons with lived experience. The goal is to create a diverse panel with people who have impact in the area of focus, representing different perspectives and knowledge. Professional and non-professional roles are valued equally as experts. Co-creation increases the probability of successful adoption and engages those with expertise and experience impacting non-medical drivers of health.

Over the course of 3–4 meetings facilitated by the DH ECHO program manager and education director, the planning team establishes: the key audiences, learning objectives, curriculum design, speaker selection, and marketing and outreach strategies to reach intended audiences. Planning meetings are scheduled to be completed 2–3 months before course launch to provide enough time for marketing, course promotion and securing of relevant continuing education credits when indicated.

Community-focused ECHOs are promoted by leveraging existing networks including community and regional networks identified by the planning team, extending reach into the community. The DH Project ECHO team disseminates information about upcoming ECHOs through past attendee lists, health system marketing lists, and a variety of organizational and professional contacts as appropriate to the topic. Community experts, including persons with lived experience, identify the networks they interface with and courses are promoted through those networks. This encourages dissemination deep into relevant communities that are often beyond the reach of traditional health system sponsored education. Registration is open to anyone and courses are always offered free of charge.

Course registration data illustrates the diverse community roles attracted to this tele-education model. The broad array of participants is illustrated in an example of registration data from a series of four ECHO courses all focused on addressing youth mental health challenges in schools and the broader community (Table 4). The diverse audience creates a rich environment for cross-professional and cross-sector learning.

**Table 4:** Self-identified roles of registrants from four community focused ECHO's courses on youth mental health .

Role	Count (%)
School Nurse	80 (18%)
Nurse (healthcare, public health, unspecified)	52 (11%)
School counselor	54 (12%)
Teacher	35 (8%)
Librarian	32 (7%)
Principal	28 (6%)
Social Worker	26 (6%)
Education Administrator	17 (4%)
Health Educator	17 (4%)
Behavioral/Mental Health Provider	16 (4%)
Director/Administrator of Community Organization	15 (3%)
School Psychologist	14 (3%)
Other Role in Education (unspecified)	11 (2%)
Care Coordinator	10 (2%)
Medical Professional	6 (1%)
Counselor	5 (1%)
Attorney	5 (1%)
Child Welfare	5 (1%)
Community Worker	5 (1%)
Religious Leader	5 (1%)
Healthcare Administrator	4 (1%)
Athletic Coach/Camp	4 (1%)
Psychologists	4 (1%)
Parole and Probation Officer	3 (1%)
Total	457 (100%)

All courses offer certificates of attendance to those who request them. Courses planned for audiences that are expected to include professionals who require continuing education, including many of our community ECHOs, offer continuing education units, most commonly continuing medical and/or nursing education credits (CME and CNE). Attendees track their session attendance at the online ECHO platform and then submit their CEU request for hours attended at the end of the course.

### 4.4 Course production

Despite differences in participant groups and course topics, the operations infrastructure and basic course production of community-facing ECHO course sessions is similar to that of clinical ECHO courses. The experience of the DH operations team provides a foundation for success, the session flow often follows the traditional, clinical ECHO model, and enduring materials are posted and circulated following the sessions.

Community-facing ECHO courses benefit from using the DH personnel, processes and technology that have been developed over years.

Perhaps the most important role in community-facing ECHOs is that of facilitator. This individual creates a welcoming and inclusive space, honoring the equal importance of all voices, encouraging inquiry, and diverse viewpoints. The diversity of participants can stymie discussion unless the facilitator nurtures collaborative learning. Our program team identifies facilitators for community courses who can create an inclusive learning environment in which power dynamics are dismantled and people feel empowered to share experiences. Facilitators may be a member of the DH ECHO team or someone trained in ECHO facilitation by the team.

Sessions begin with participants welcomed to the course by the facilitator, oriented to the principles and philosophy of the all teach, all learn ECHO model, and encouraged to share freely through the chat function and in oral discussions. This onboarding provides a shared understanding of the ECHO model and processes. Technology support is provided by the DH ECHO team at each session.

Invited speakers provide a short didactic, generally lasting only 15–20 min of the hour session. After clarifying questions for the speaker, a relevant case or case proxy as described below is presented, followed by facilitated conversation. Participants ask questions, share their perspectives, and the virtual group engages in collaborative learning as they use “real world” experiences to address the presented challenge. While the emphasis is on shared learning cultivated from the wisdom and experience of participants, a multi-perspective panel of experts also attends each session to share their expertise in the topic being discussed. The expert panel usually includes persons who served on the planning team, but may also include additional people with knowledge in the area of interest. Experts are not defined by their professional roles but rather by the depth of knowledge and experience they bring to the subject matter; persons with lived experience bring critical expertise to each course.

Case-based learning is a foundational component of both clinical and community ECHO courses. In community-facing ECHOs, the definition of cases has been broadened from clinical case presentations to include other devices to stimulate interactive discussion and learning. These include: presentations that describe a common system level barrier encountered by community organizations, role playing scenarios, or the use of participant polls that explore different options for addressing a common challenge.

Common to both clinical and community-focused ECHO courses, materials are curated and posted on the DH ECHO Hub pubic website, available to anyone regardless of participation. Postings usually include audio-visual recordings of the didactics and slide sets (rarely a speaker declines sharing materials), case discussion notes, and course resources lists compiled from speaker slides and contributions from participants and panelists during the discussion. The DH ECHO team occasionally supplements resource lists based on research done in response to issues raised during the course. ECHO community-facing course resources remain posted for up to five years before being archived, as long as they remain generally relevant and appropriate to current practice.

### 4.5 Evaluation and reflection

Courses are evaluated using the same approach for both DH clinical- and community-focused ECHO courses. Participants are asked to complete surveys prior to the first session of a course and after the course is completed. DH ECHO operations staff and course-specific team members meet together once survey data has been compiled to reflect on successes and challenges with the course. These lessons learned are applied to the design and implementation of future courses, an important implementation construct described in the CFIR model.

The RE-AIM model emphasizes measures of effectiveness when implementing programs. The number of community-focused courses produced each year, number of registrants, and average number of attendees at each session are used as global measures of effectiveness. In the first year producing community-focused courses, there was an average of 80 registrants per course. In the most recent year, that number had increased to 180 registrants per course. Additionally, DH ECHO program staff monitor the registration and self-identified roles (Table 4) to evaluate success reaching the intended audience. Previous authors have used similar metrics to evaluate effectiveness of ECHO programs (30).

At the participant level, course evaluations include individual survey questions presented in the pre-course and immediate post-course periods and ask participants to rate their knowledge and/or confidence related to the learning objectives of the course on a 0–5 Likert scale (0 = no confidence; 5 = highly confident). The immediate post-course survey also asks about anticipated changes in practice and behaviors as a result of participation in the course, and queries participants if they have experienced a decrease in professional isolation as a result of attending the course. Surveys sent 3-months after the course ask what actual change the participant has adopted and again ask about reductions in professional isolation. All surveys include open text responses which are a rich source of suggestions, criticisms, and compliments.

**Figure 1:**
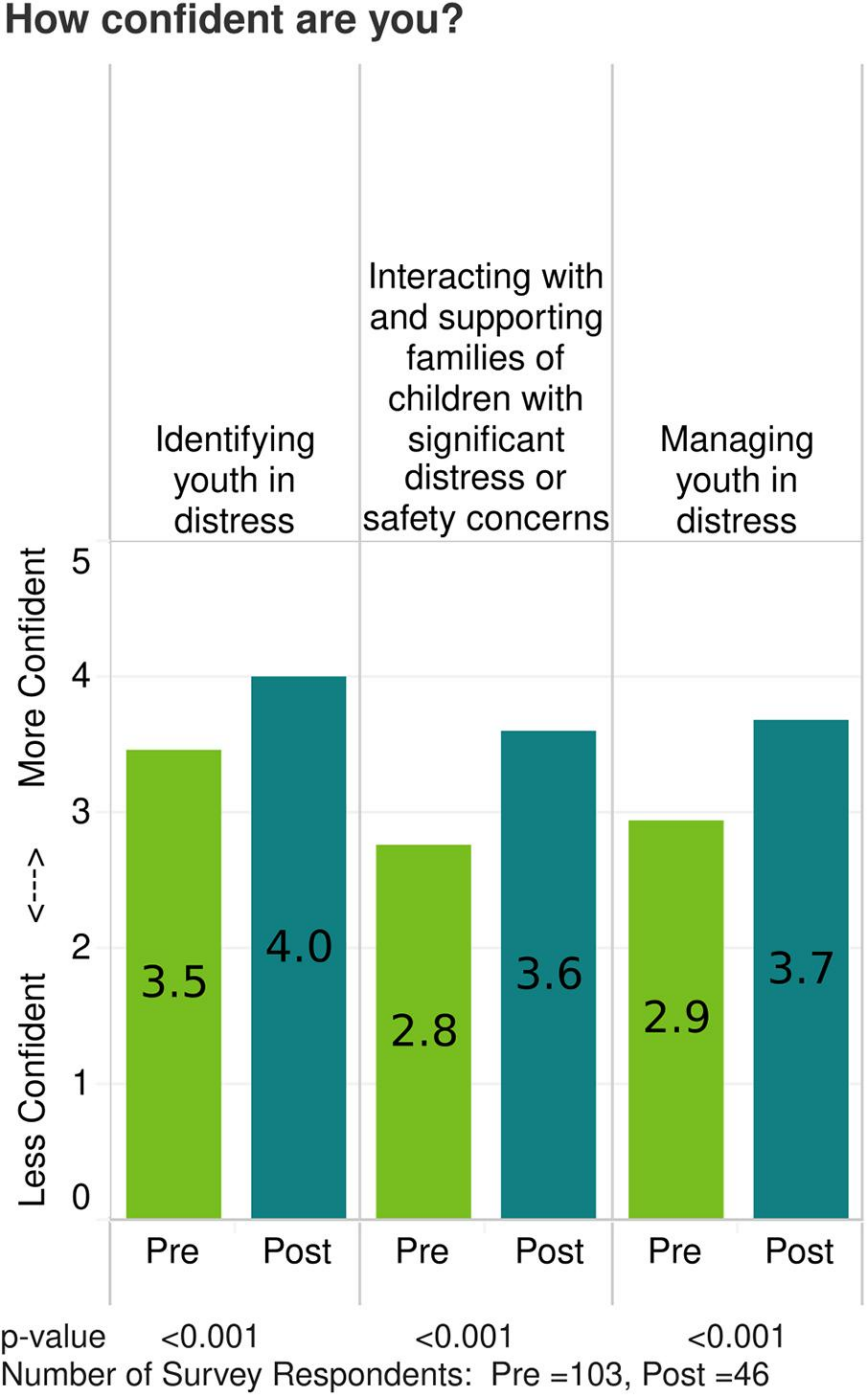
Example of change in mean confidence in course objectives self-reported by participants on pre- and post-course surveys. Data is from two youth mental health ECHO courses that primarily engaged school personnel.

Detailed analysis of participant survey results across the 36 community-focused courses is beyond the scope of this article however, an example of a single course evaluation, Keeping Students Safe, is included as a supplement. Change in confidence is calculated using unpaired t-tests for each survey question. Figure 1 demonstrates pre/post-survey results for a series of two community-focused ECHO courses focused on providing school staff with knowledge and skills to support youth with mental health concerns. For all community-focused courses in which analyses have been completed, there have been significant increases in confidence in pre- to post-survey questions.

Actual changes in health outcomes and health equity have not been attributed to the DH ECHO courses. Across all courses, between 80% to 100% of course attendees report they feel less professional isolation as a result of attending a community-facing course; developing a community of learning may be an important contributor to improvement in the long run.

## 5 Discussion

We believe this is the first paper to describe ECHO program adaptations to produce courses that focus on impacting community conditions that are major drivers of health outcomes. The DH ECHO Hub has adapted traditional, clinically-focused ECHO programming to produce courses focused on “upstream” determinants of health. These community-focused ECHOs bring diverse sectors of our communities together in collaborative learning sessions to increase knowledge about political, socio-economic, behavioral, and structural drivers of health and explore opportunities for community members to change community conditions that impact health.

In this paper, we retroactively applied the RE-AIM and CFIR frameworks to describe the implementation of our community-facing ECHO programming (Table 2). CFIR describes conditions that may impede or facilitate implementation (26) and many of our implementation activities mapped to CFIR domains. The first step when considering production of a community-focused course is to evaluate the advantage adapting the traditional ECHO model compared to other learning models such as podcasts or webinars, constructs outlined in the CFIR innovation domain. Consideration of community conditions (outer setting), existing health system capacity and DH ECHO infrastructure (inner setting), and the role of community members co-designing courses (individuals' domain) are all important conditions impacting our community-facing ECHO programs.

Elements of the RE-AIM framework also resonate with our experience (33). Selecting courses for ECHOs includes an assessment of the alignment between the topic and the goals and priorities of the health system; close alignment increases the likelihood of sustained community-health system relationships after the course is finished (maintenance). Including members of the community as partners in course design and implementation increases adoption and reach. Utilization of existing health system infrastructure and ECHO processes, including the established evaluation processes developed for clinical ECHO courses, has strengthened implementation and effectiveness.

Dissemination and implementation frameworks have been applied to ECHO programs to guide implementation, evaluate program offerings, and assess organizational readiness for implementing ECHO programs (22, 26, 30). Notably, the majority of ECHO programs evaluated in the literature were designed to improve access to high quality health care services by improving providers knowledge and skills in specialty areas. The DH ECHO experience designing and implementing population health-focused programs suggests that these frameworks may be useful for replicating telementoring programs focused on the upstream determinants of health.

The effectiveness of the ECHO model and similar collaborative, tele-education models has been the subject of numerous reviews with growing evidence of impact at the provider and patient level (23–25, 29). The rapid uptake and dissemination of the model suggest this is a highly valued tele-education method, even though multiple reviews raise questions about the effectiveness of the model to improve outcomes and universally recommend additional studies to evaluate impact (24, 25, 29, 31). Over the 5 years we have produced community-focused ECHOs, average registration per course has more than doubled suggesting our courses are valued by our communities.

Our paper describing an ECHO model to improve population health outcomes has several limitations. We describe the experience at a single health system and results may not be generalizable. Important characteristics of our context may be unique to our program and inhibit broad dissemination. However, the implementation frameworks applied to our program may offer guidance to other ECHO Hubs considering adapting their programming to impact the social determinants of health.

The impact of DH community-facing ECHOs on population health is unknown. Our measured outcomes, indicate significant improvement in participant confidence around stated learning objectives but are limited to these subjective variables and do not have capacity to measure outcomes at the community health level.

We are encouraged by our participants self-reported changes resulting from participation in our courses but it is unknown if this will translate to ongoing, multi-sectoral collaborative work to improve upstream drivers of health.

## 6 Conclusion

Many sectors of society will have to work together to improve the health of the US population. Health care delivery systems need to re-examine their roles to improve health outcomes, not just health care services. Academic health centers need to explore innovations in education and training methods to meet the challenges ahead. The DH ECHO Hub adapted the ECHO model with the goal of engaging diverse members across our communities in education that would empower them to impact the upstream conditions that drive health outcomes and health equity. Examining our community-focused ECHO course implementation process with the CFIR and RE-AIM frameworks, our processes mapped to multiple domains in each framework. Implementation frameworks may inform ECHO hubs and other virtual education providers how to adapt education models to meet the learning needs of communities and health systems as they address the complex drivers of health in our communities.

## Data Availability

The raw data supporting the conclusions of this article will be made available by the authors, without undue reservation.
