# Supplementary material for: Lessons learned implementing an innovative extension for community healthcare outcomes (ECHO) program
Source: Front Health Serv. 2026 Jan 12;5:1682447. doi: 10.3389/frhs.2025.1682447 (PMC12832770; doi:10.3389/frhs.2025.1682447)
Supplement: Supplementary file 1 [file Datasheet1.pdf]

Project ECHO at Dartmouth Course Summary for QI and Archival purposes

Course Title ____Keeping Students Safe: Recognizing and Responding to Youth in Distress ECHO

Learning Objectives

1. Articulate the principles and values of peer support
2. Understand the training and credentialing process requirements of how peer support works
3. Describe the 5 component model of peer support supervision
4. Recognize common challenges faced by peer supporters in the community
5. Implement supervision of peer support workers in alignment with guidelines for peer support

Session Dates, Topics and Speakers

Dates	Topics	Speakers
5/2/23	Recognizing youth in distress	Kattie McKinnon, MA
5/16/23	What might we be seeing - the underlying causes	Chase Trybulski , LCMHC
5/30/23	What can we do about it? Building resiliency and connection	Becky Parton, MSW,LICSW
6/13/23	Getting help when we need it-resources and working with families	Mark Belanger
6/20/23	Practical strategies for this moment in time	Mark Belanger

Panelists:

Name and degrees	Perspective-Profession	Regular or Visiting
Jackie Pogue, MA, MS<br>Julie Balaban, MD	Facilitator<br>Course Director	Regular<br>Regular
Barbara Dieckman, RN,MS,MBA	Co-Director	Regular
Mark Belanger, MBA	Consultant	Regular
Steve Beals	Principal, Alvirne High School	Regular
Lisa Hayward, PhD	NH DOE Office of Social and Emotional Wellness	Regular

Becky Parton, MSW, LICSW	Project Director, Dartmouth Trauma Interventions Research Center	Regular
Rabbi Robin Nafshi	Rabbi Temple Beth Jacob	Regular

Marketing and circulation venues

- Sent to previous participants in past ECHOS
- Planning team distributed to their mailing list

Participant information

- Number Registered- 114
- Number registered from D-HH system - 15
- Avg session attendance – 50

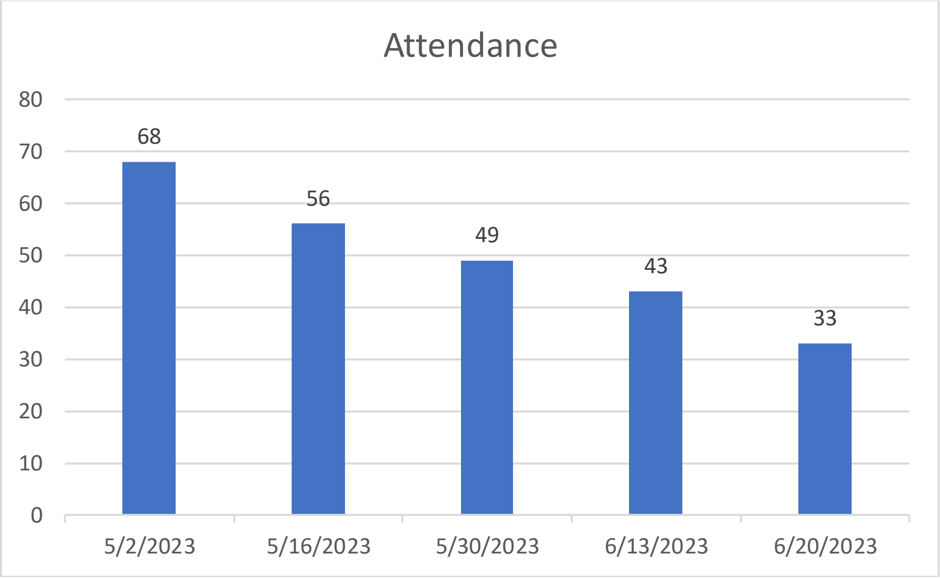

Graph of recording views

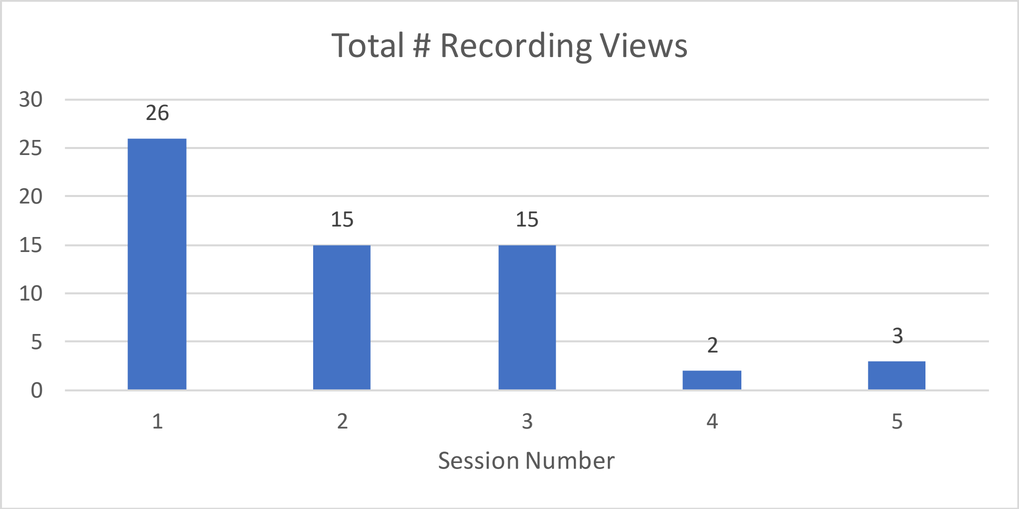

ECHO Participant Demographics Total Registrants: # 114

Principals/other roles in education	21
Librarians	18
Health Education coordinators	16
Nurses	15
Administrators/Directors	11
Athletic coaches + Camps	4
Legal	4
Child Welfare	3
Psychologists/School Psychologists	3
Other	19

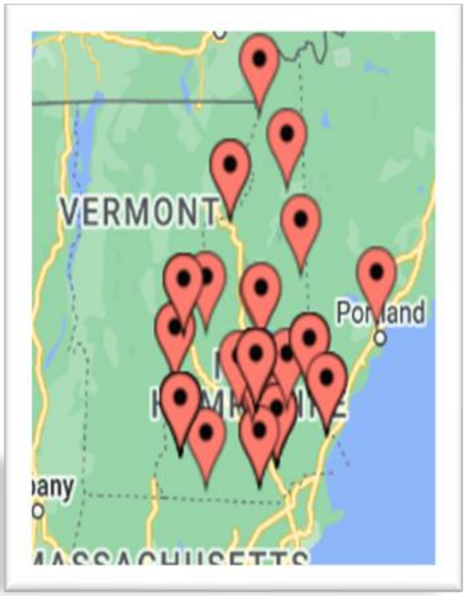
VERMONT
Portland
Albany
MASSACHUSETTS

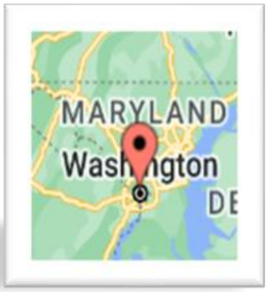
MARYLAND
Washington

Outcomes Data

Participant confidence measures pre & post (graph)

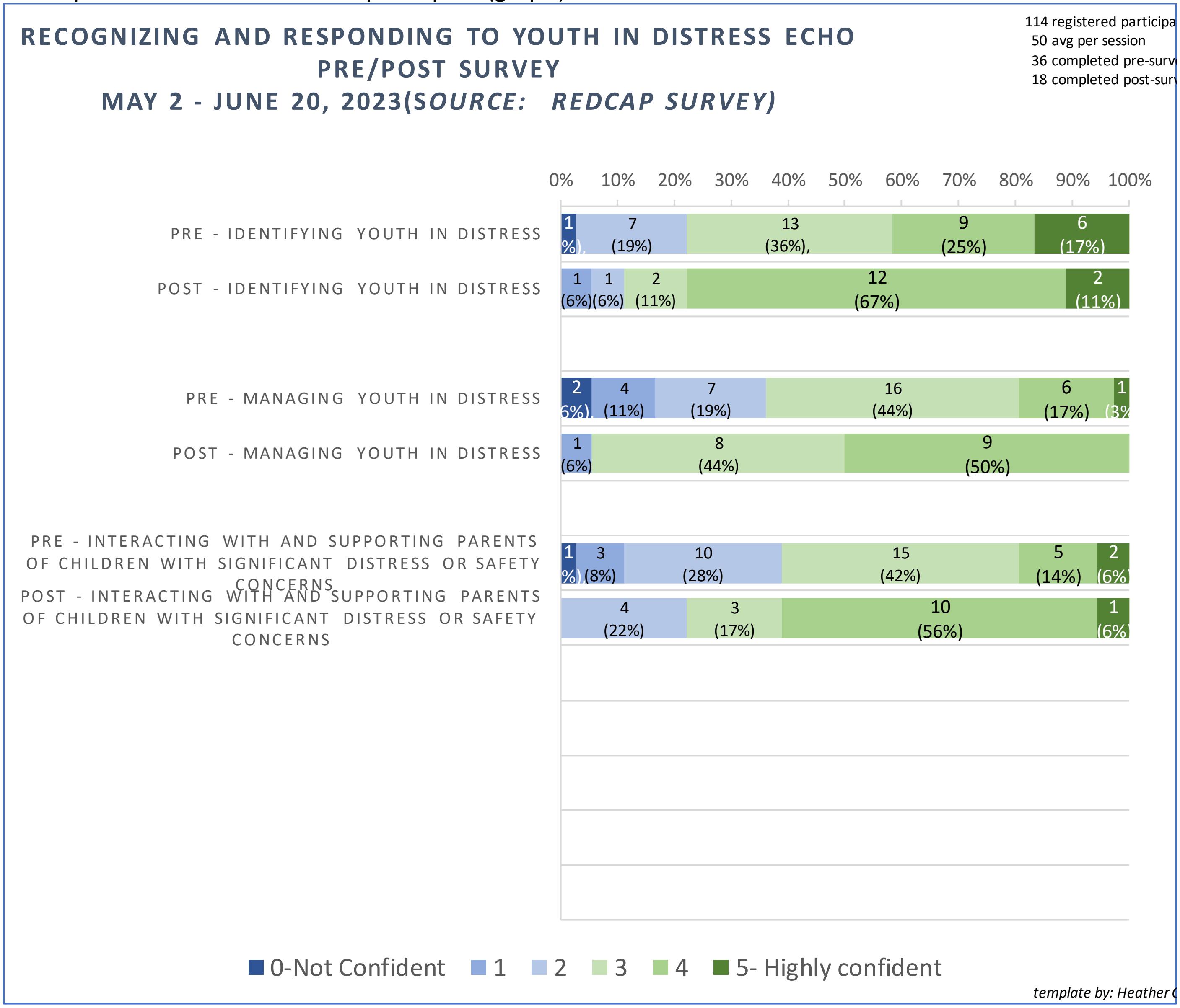

Overall Confidence Scores- Pre: 68%, Post: 87%

100% of participants felt a decrease sense of professional isolation after attending this ECHO.

Narrative examples of anticipated practice changes

- I would like to gain confidence in working with youth that are in crisis, especially teens.
- To be able to identify youth in distress, who to tell when I suspect someone is in distress and then how to be supportive.

- I would hope to be able to identify a child in distress and to let a child know we are a safe place for them
- Being able to identify and help children in distress.
- Ideas/suggestions to help in my role as librarian. I also don't know what are some key factors that indicate someone is in distress. If I know the child well, I can sometimes tell...
- Best resources that I can pass along to youth in distress and their families. Training for my own verbal and non-verbal responses when managing youth in distress. How to interact with parents in a fair manner.
- I have limited interaction with minors in my practice dealing with HIV. some are too young to understand their status, others are teenagers who think they are immortal and don't need to take their meds. I'm hoping to find ways to better connect to my teen patients and help them get a better understanding of their condition.
- I am primarily interested in this program because I am the Program Manager for Mental Health First Aid at UNH Extension. As part of developing new programs - I hoping to learn from what is being offered and how it presented. As a clinician who worked with youth and families for over 35 years; I am extremely interested in this topic and learning what others are doing to engage this difficult population.
- Be able to assist the providers and families I work with more knowledge than I currently have.
- To increase my skills at managing youth in distress especially when parents and teachers are apathetic.
- I would just like to be able to help in any situation that might come up when working with kids and teens at the public library.
- To learn how to identify & support distressed youth in a library setting
- Increase my knowledge in identifying and supporting youth in distress
- My hope for this program is for me to be better equipped to recognize teens in distress when working with them through our community programs. I would also hope to add to the knowledge I am learning in college.
- More knowledge of how to talk to youth and parents. What do you actual say. I don't want to ignore a situation but also don't want to make a situation seem so serious that I make a problem (one that is part of everyday life) that with some support is a lesson in resilience become one I make sound and youth and parents believe is so serious they feel there is something wrong with them.
- I want to take a more active role with guidance/admin/home to school coordinator in supporting students and families
- To identify and help children in distress.
- Follow up after the sessions where all the information / ideas are collected under the subtopic since the chat moves quickly.
- To learn more about this urgent topic, to gain tools to use in solving these problems, and to deepen a sense of community surrounding the issue.

- I hope to gain more knowledge on identifying and helping to manage youth in distress. Specifically, I would like to learn more about resources, language, working with parents, etc. Looking forward to this.
- Getting more tools to help parents and the young people I work with.
- I am looking forward to any new information shared on identifying youth at risk. Today's speaker was very informative in those topics. Thank you.
- Build confidence in supporting families with youth in distress!
- I hope to build on what I learned in the Youth Mental Health First Aid course, as well as learn strategies and skills from others who work with youth.
- Perspective of others who deal with our youth. Today's session was very eye opening as to what is going on in a library. We spend so much time and effort keeping kids safe at schools, yet set them free into an unaccounted atmosphere. It is quite interesting listening. Thank you.
- Just a general awareness of how to recognize and act on youth in distress.
- How to interact with supporting parents and staff with youth in distress
- To learn more about how to help youth in distress to the best of my ability

Additional comments

- Looking for more local resources. Liasing with local school district.
- Continuing to make sure I take Youth Mental Health First Aid every year. Continuing to form collaborative opportunities with other community partners.
- Feel more confident with language to use to keep conversation ongoing. I've identified resources and continuing training for my staff members.
- I felt like in most cases participants reiterated what the issues are and acknowledged that they are difficult to encounter, however other than session 3, there were not a lot of relatable and useable, practical tips and information given on how to handle situations. Even the cases we discussed - we never really came up with a "resolution" or suggestions on how to handle that situation (it felt more like a support group).
- I am a school nurse (K-5). There are daily protocols and so many kids who need support so I want to be more involved and support my student's guidance counselor. I hope to attend more parent meetings/wrap around sessions and be available for students if the guidance counselor is not available.
- Due to the helpfulness of this program, I'll be inviting other members of my church community to attend the Mental Health First Aid Training with me in the near future. I'll also be discussing/establishing Guidelines/Boundaries with the youth on Day 1 of our programs in the fall.
- Listening more..."being present" Being a better resource person
- I am a school nurse (K-5). There are daily protocols and so many kids who need support so I want to be more involved and support my student's guidance counselor. I hope to attend more parent meetings/wrap around sessions and be available for students if the guidance counselor is not available.

- More awareness about the role of community liaisons as a resource for reducing youth distress (e.g., librarians).
- Taking a youth mental health course. Liaising with local school districts for training purposes and coordination of services.
- Working harder with community supports. Maybe attend libraries to see what issues arise and maybe collaborate with them and our district after school program.
- I will look into NAMI training as well as researching more about local organizations that may be helpful resources. I will also download all resources provided by the weekly speakers to use as a reference.
- Being more alert of our youth when being around them. Trying to identify triggers and warning signs.
- I will be posting some of the information presented in common areas and also sharing those resources with other staff members.

Other potential topics

- Being queer in NH; staff mental health/burnout
- How to motivate young people - how to help them understand that hard work is important and will help them in the long run. Too many students do "just enough" to get by and don't apply themselves and try to learn as much as possible.
- child abuse identification and reporting
- Boundary setting. How to continue teaching and learning within a group in the face of individual provocative, dramatic and pessimistic statements.
- child abuse identification and reporting
- Navigating community resources. Trauma support.
- More topics that can involve librarians!
- This was my first ECHO course. I found it to be very enriching. I will keep my eyes out for future topics, as most anything related to youth pertains to my role within the school.
- Discussing the importance and approach of advanced sex ed in junior high and high school.
- Good question, but I'm not sure at the moment. The presentations were informative and well-presented.
- I would love to have more of an opportunity to hear from those in the medical field who receive patients and conduct mental health screenings, etc. I am always curious what patients (particularly school students) share as their reasons for feeling certain ways and if that dialogue between medical professionals and teachers/parents, for instance, could help shape our curriculum in schools as well as the ways in which we respond to our students/children.

Total responses from panelists: 4

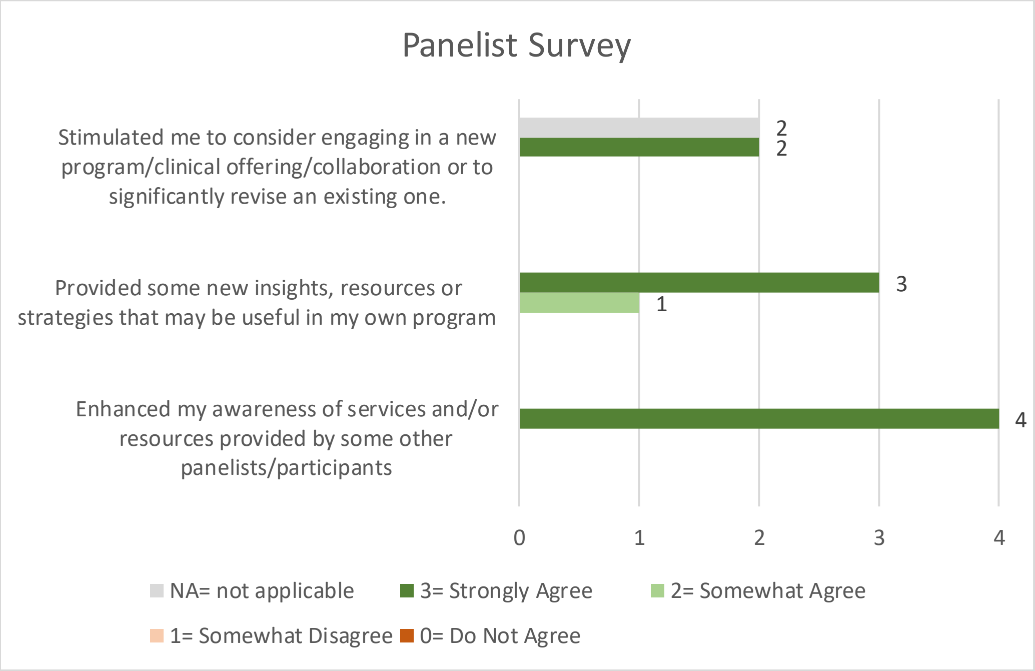

Panelists comments:

- I enjoyed the session and the different professionals that were attracted to it. I felt the library personnel dominated the session, in a positive way and hope we may do more in the future with others who also work with children.

Three month post course survey data: Added 11/14/2023
Total Responses: 10

90% of participants felt a decrease sense of professional isolation after attending this ECHO.

Practice Change

- More aware. Better tools
- I'm more aware of community resources and asking how the school district can connect with and distribute those resources.
- I never realized how others, libraries/librarians, are impacted. My understanding broadened quite a bit with the course.

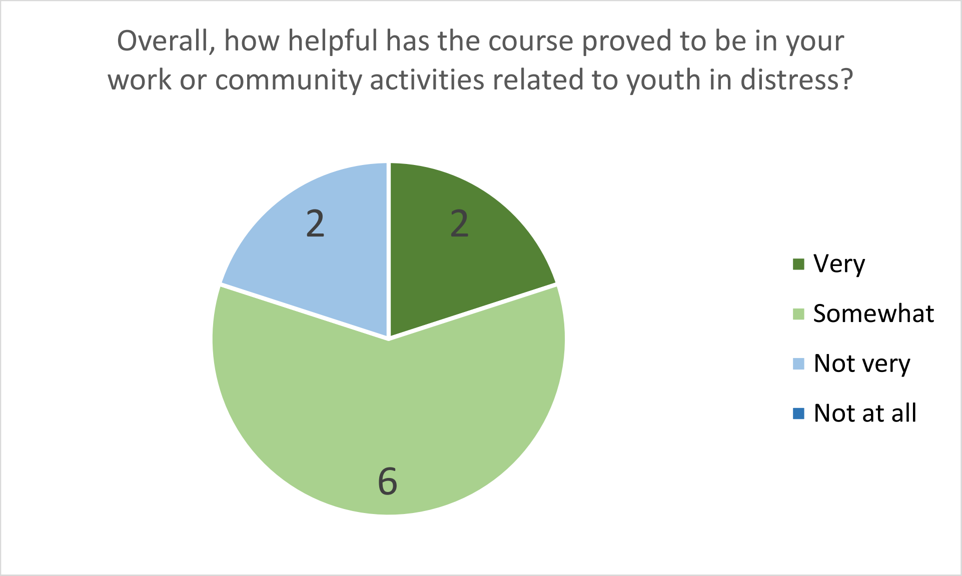

Suggestions for changes to future courses:

- I appreciated the prompts and engagement from others across the state. I think each topic/question went on a bit too long. Less talking/input about each will give us a chance to hear from more people.
- More very specific guidance. It would be more helpful to have scripts or procedures dictated by someone who knows what they're talking about and then to discuss the ways to adapt it to our unique setting.
- The case study thing was limited and kind of annoying. I valued the experts more

Recommend this course to others? 78% (7/9) said Yes

How much did having a mix of professions enhance this program?

- I think it was amazing to hear other professionals opinions.
- I enjoyed the Project ECHO team and how diverse it was. Great to hear from so many principals, educators and librarians (who knew!)
- Helpful to see variety of perspectives and solutions.
- Greatly! it was so informative.
- A lot! It was really nice to her from totally different perspectives. Sometimes we get tunnel vision and I really appreciated hearing other ideas.
- I think it provided some good food for thought, but it would be helpful also to establish a baseline of professional practices/standards for each of the represented professions.
- it was fine
- It was extremely helpful to look at the same issues from another vantage point.
- I enjoyed hearing the multiple perspectives, the limitations and challenges in different sectors.

- Very helpful to hear from other view points and realizing the other groups that are trying to address this - that could be resources.

How much did having a mix of professions detract from this program?

- It did not detract it enhanced it.
- There were some professions involved that were just not dealing with the same things I see in my every day work. I appreciated the diversity though.
- None
- Not at all in my opinion.
- Sometimes people would spend a lot of time giving advice or suggesting solutions that we've already tried and which failed on our library setting.
- Perhaps it didn't yield targeted results from the session, but I was able to bring new ideas to my cohort. I guess it didn't detract at all.
- Often, it felt like the information did not pertain to me.

Additional comments:

- Thank you for hosting.
- I found it off-putting
- Looking forward to the next course.

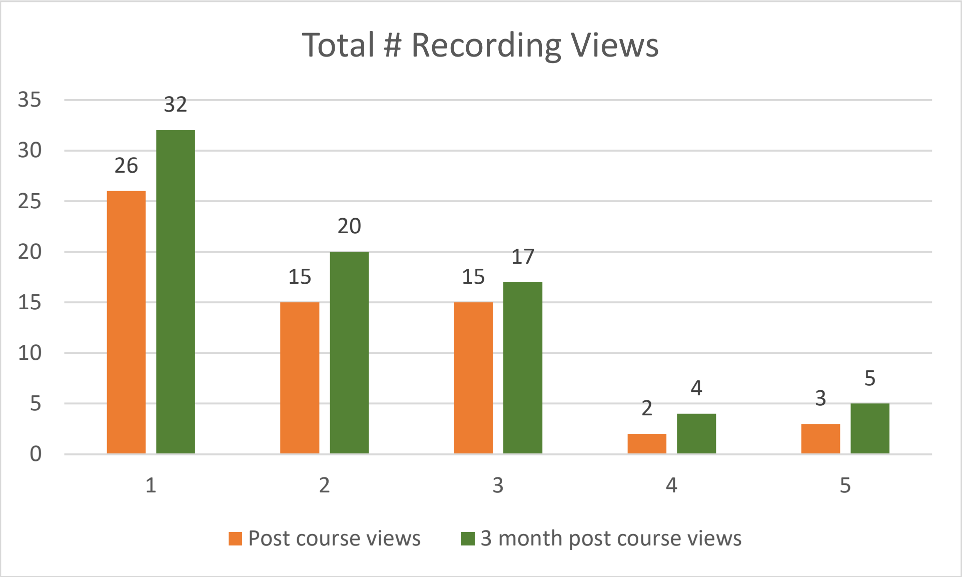

Post course average views: 12
3 month post course average views: 16
